# Cognitive Reserve as a Protective Factor of Mental Health in Middle-Aged Adults Affected by Chronic Pain

**DOI:** 10.3389/fpsyg.2021.752623

**Published:** 2021-10-25

**Authors:** Selma Delgado-Gallén, M. Dolors Soler, Sergiu Albu, Catherine Pachón-García, Vanessa Alviárez-Schulze, Javier Solana-Sánchez, David Bartrés-Faz, Josep M. Tormos, Alvaro Pascual-Leone, Gabriele Cattaneo

**Affiliations:** ^1^Institut Guttmann, Institut Universitari de Neurorehabilitació adscrit a la Universitat Autònoma de Barcelona (UAB), Badalona, Spain; ^2^Departament de Medicina, Facultat de Medicina, Universitat Autònoma de Barcelona, Bellaterra, Spain; ^3^Fundació Institut d'Investigació en Ciències de la Salut Germans Trias i Pujol, Barcelona, Spain; ^4^Universitat Autònoma de Barcelona, Bellaterra, Spain; ^5^Institut d'Investigacions Biomèdiques August Pi i Sunyer, Barcelona, Spain; ^6^Departament de Medicina, Facultat de Medicina i Ciències de la Salut, Universitat de Barcelona, Barcelona, Spain; ^7^Hinda and Arthur Marcus Institute for Aging Research and Center for Memory Health, Hebrew SeniorLife, Boston, MA, United States; ^8^Department of Neurology, Harvard Medical School, Boston, MA, United States

**Keywords:** cognition, disease susceptibility, middle-aged, healthy lifestyle, brain reserve, psychological distress

## Abstract

Chronic pain is associated with worse mental health and cognitive impairment, which can be a cause or a consequence of brain structure and function alterations, e.g., maladaptive plasticity, antinociceptive system dysregulation. Cognitive reserve reflects the effectiveness of the internal connections of the brain and it has been shown to be a protective factor in brain damage, slowing cognitive aging or reducing the risk of mental health disorders. The current study explored the impact of chronic pain on psychosocial factors, mental health, and cognition. Furthermore, we aimed to examine the role of cognitive reserve in the relationship between mental health and chronic pain clinical characteristics in middle-aged adults. The study group consisted of 477 volunteers from the Barcelona Brain Health Initiative who completed online surveys on pain, mental health, cognitive reserve, and psychosocial factors (sleep and quality of life). We described the differences in sociodemographic data, psychosocial factors, mental health, and self-perceived cognitive impairment, and neuropsychological assessment, between participants reporting pain compared with those without pain, as well as the main characteristics of the chronic pain group. Finally, to study the role of cognitive reserve in the modulation of the relationship between chronic pain and mental health, we compared variables between subgroups of participants with high/low pain intensity and cognitive reserve. The results showed that chronic pain was reported by 45.5% of middle-aged adults. Our results revealed that participants with chronic pain were older and had worse health status than people without pain. The presence of chronic pain affected working memory, mental health, and daily life activities. Moreover, cognitive reserve moderated the influence of pain intensity on mental health, resulting in less mental health affection in people suffering from high pain intensity with high cognitive reserve. In conclusion, the construct of the cognitive reserve could explain differential susceptibility between chronic pain and its mental health association and be a powerful tool in chronic pain assessment and treatment, principally due to its modifiable nature.

## Introduction

Chronic pain, a pain persisting beyond 3 months, has been linked with altered mood and mental health disorders (Baliki and Apkarian, 2015; Baker et al., 2016; Kawai et al., 2017), cognitive impairments (refer to Moriarty et al., 2011 for a review), sleep disturbances (Karaman et al., 2014; Generaal et al., 2017), and decreased quality of life (Leadley et al., 2014; Kawai et al., 2017; Senba and Kami, 2017).

These factors can influence the experience of pain and help the transition from acute to chronic pain (CP) (Apkarian et al., 2013). Furthermore, pain is strongly influenced by the cognitive context (Kucyi and Davis, 2015), altering the response to subsequent noxious stimuli, which potentially contribute to CP development.

A complete model for understanding pain must include not only risk factors, which have been widely studied but also protective ones. In this regard, Cognitive Reserve (CR) could potentially play a role. CR is intended as the adaptability of cognitive processes that explain differential susceptibility to brain aging, pathology, or insult (Stern et al., 2020). People with higher CR have more efficient brain networks that allow them to actively cope with pathology using compensatory mechanisms (Stern, 2002, 2009; Barulli et al., 2013). Previous studies suggested the role of CR as a protective factor in dementia and traumatic brain injury (Reynoso-Alcántara et al., 2018) or its moderator effect in psychiatric diseases (Pertl et al., 2017; Opdebeeck et al., 2018), but only a few studies explored CR associations with CP and emotional factors. The study of Gómez-Beldarrain et al. found that high levels of CR in migraine patients, could facilitate pain control and reduce the tendency to migraine chronification (Gomez-Beldarrain et al., 2016). On the other hand, low CR estimates decreased quality of life, increased anxiety and depression, and increased risk for drug dependence and medication overuse (Gómez-Beldarrain et al., 2015).

Given these results and considering the importance of cognitive processes in pain chronification reported in previous literature (Bushnell et al., 2013; Landrø et al., 2013; Attal et al., 2014; Moriarty and Finn, 2014; Brucki and Brucki, 2016), we hypothesized that CR could modulate cognitive processes which can vary its impact on mental health (MH) in the presence of CP.

The aims of the current study were: (1) to investigate the presence of CP, its clinical characteristics, and their impact on measured and self-perceived cognition and general psychological aspects (sleep and quality of life), (2) to explore its putative interference with specific psychological aspects related with MH, and (3) to examine the hypothesized CR protective role, expected to weaken the relation between pain and MH in a healthy middle-aged sample.

## Materials and Methods

### Participants and Study Design

This study involved 477 healthy voluntaries from the Barcelona Brain Health Initiative (BBHI) (Cattaneo et al., 2018, 2020), who answered online questionnaires (Phase I) and performed an in-person neuropsychological assessment (Phase II). BBHI is an ongoing prospective longitudinal study that started in 2017 to identify lifestyle factors and biological mechanisms underlying good brain health in healthy middle-aged adults (between 40 and 65). The main BBHI aim was to study healthy participants in a pre-clinical stage of possible age-related pathologies, like dementia or Parkinson's disease. Due to this, the inclusion criterion was to be aged between 40 and 65 years (middle-aged adults) and to recruit only healthy participants. Exclusion criteria were if they had a history or current diagnosis of neurological disease, traumatic brain injury associated with post-traumatic sequelae, or current or recent diagnosis (<2 years) of psychiatric conditions, e.g., major depression, post-traumatic stress disorders, substance abuse/dependence.

**Figure 1:**
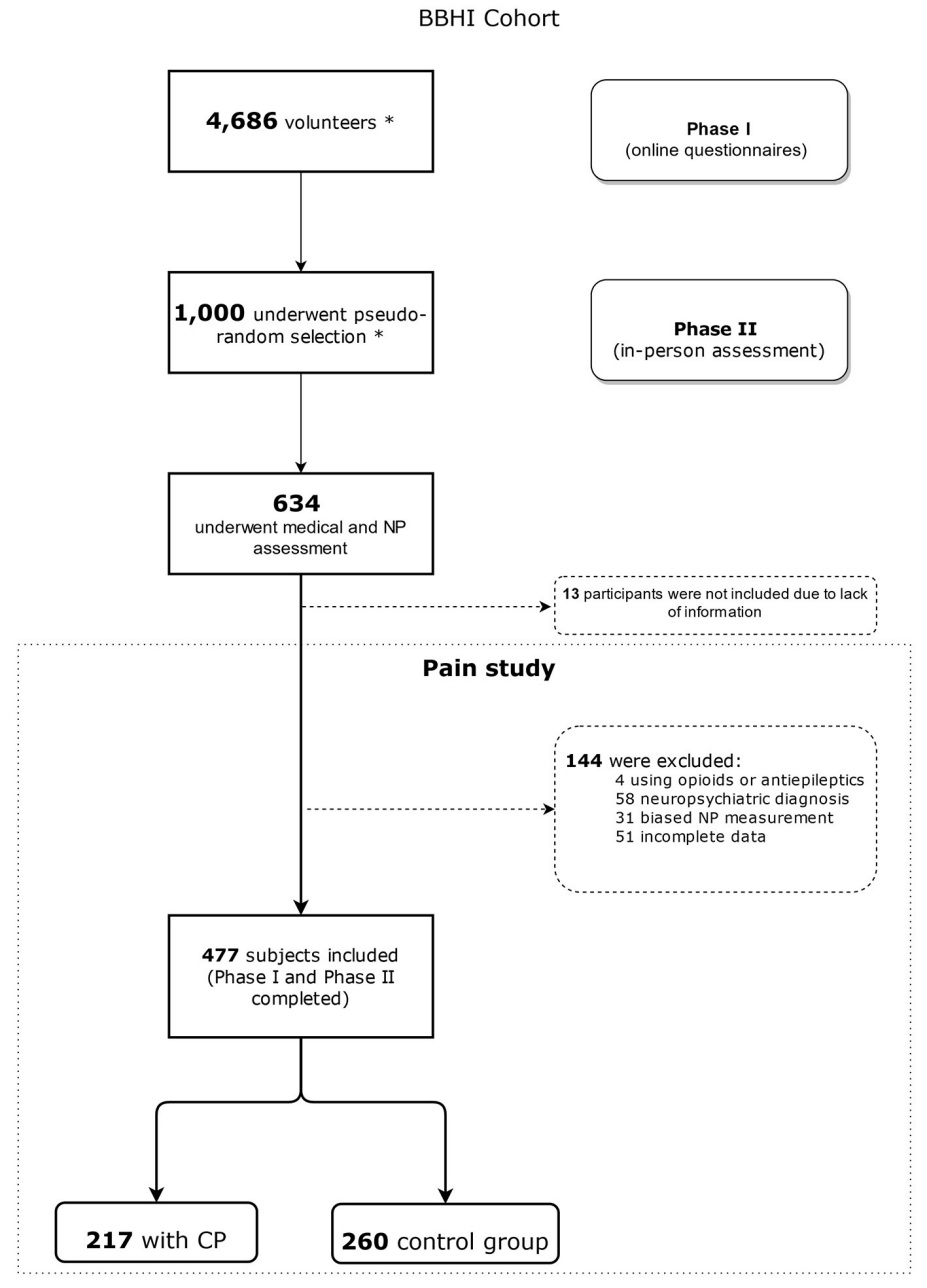
Flowchart of the selection and distribution of patients in this study. BBHI, Barcelona Brain Health Initiative; NP, Neuropsychological; CP, Chronic Pain. ^*^Volunteers were selected by BBHI methodology. For more detail information refer to Cattaneo et al. (2018, 2020). For this study, we selected participants enrolled in the project until February 2020.

Participants were divided into two groups based on CP presence/absence (as shown in Figure 1), i.e., if they had been having pain for the last 3 months.

The protocol was approved by the “Comité d'Ètica i Investigació Clínica de la Unió Catalana d'Hospitals” and was carried out following the Declaration of Helsinki (World Medical Association, 2013). Written informed consent was obtained from all participants before inclusion in the study.

#### Online Assessment

In Phase I, 4,686 participants underwent an online self-assessment between July and December 2017 through the BBHI web-based platform about sociodemographic characteristics, MH, CR, and psychosocial factors (sleep, quality of life, and self-perceived cognitive impairment), as well as a detailed screening for CP or other chronic conditions.

#### In-person Assessment

In Phase II, from May 2018 to February 2020, 634 participants selected from 1,000 performed an in-person assessment, including a medical and physical exam and a cognitive assessment.

The same evaluation protocol was applied to all participants. First, participants underwent a medical visit with a physician (SA), which included a clinical interview to assess previous and present medical conditions, body mass index (BMI), presence of CP and medication intake, tobacco, alcohol (Alcohol Use Disorder Identification Test; AUDIT) (Carretero et al., 2016), and use of other substances. A neuropsychological assessment session was conducted by two expert clinical neuropsychologists (CPG and VAS). The neuropsychological tests (as shown below) were administered in the same order for all participants.

#### Inclusion and Exclusion Criteria

At the time of this analysis, between the 4,686 participants who completed the baseline set of Phase I questionnaires (sociodemographic, psychological aspects, psychosocial factors, pain-related questionnaires, etc.), we selected 477 subjects who also participated and completed Phase II (neuropsychological and medical in-person assessment). Thus, our sample underwent completely both the online (surveys) and the in-person assessments (neuropsychological testing).

The main exclusion criteria, as explained above, was to have a diagnosis of neurological disease, traumatic brain injury associated with post-traumatic sequelae, or current or recent diagnosis (<2 years) of psychiatric conditions, e.g., major depression, post-traumatic stress disorders, substance abuse/dependence. Moreover, participants currently consuming opioids or anti-epileptics drugs, which can affect cognitive performance, and participants with incomplete or neuropsychological assessment biases, e.g., interruption during the assessment, previous knowledge about tests, or cultural background, were excluded from the analyses.

### Assessments

#### Online Surveys

##### Pain

Participants were screened online for CP, which was considered the pain that persists or recurs for >3 months according to the International Association for the Study of Pain (IASP) criteria (Treede et al., 2015). Only those participants who answered to have recurring or persisting pain underwent the posterior pain assessment explained below.

The Brief Pain Inventory-Short form (BPI-SF) (Keller et al., 2004) was used to assess the intensity and severity of pain and pain onset. Validation studies among CP patients, and the published Spanish translation demonstrated good psychometric properties (Badia et al., 2003).

Individuals referring presence of CP were asked to indicate the location of pain on the body map that was modified to adapt to online administration. To answer pain-related questionnaires, participants were asked to identify the most prevalent/disturbing pain, i.e., participants with more than one pain site selected the main pain location. In addition, they were told to answer how many sites or kind of pain/location they had (one or more than one site). The pain location categories included: headache, facial pain, neck, low back, visceral or pelvic pain, joint pain, e.g., knees, shoulders, and chronic widespread pain.

##### Depression and Anxiety (MH)

We employed the ultra-brief self-reported Patient Health Questionnaire-4 (PHQ-4). It is a screener for anxiety and depression, which combines the Patient Health Questionnaire-2 (PHQ-2) and the Generalized Anxiety Disorder-2 (GAD-2). The PHQ-4 had four items asking mood disorder symptoms (two items for depression and the other two items for anxiety) in the past 2 weeks. All items were rated on a 4-point scale ranging from 0 (not at all) to 3 (nearly every day). Given that the depressive and anxiety symptoms were highly correlated (*r* = 0.71), a sum score of all four items was used to represent psychological distress. In addition, we divided responses into four psychological distress levels, i.e., normal (0–2), mild (3–5), moderate (6–8), and severe (9–12) according to the diagnosis guideline suggested by Kroenke et al. (2009). The published Spanish translation also demonstrated good psychometric properties in a validation study (Kocalevent et al., 2014).

##### Cognitive Reserve

Cognitive reserve was assessed using a short questionnaire (Cognitive Reserve Questionnaire; Rami et al., 2011) evaluating classical proxies of CR like the attained level of education, occupation, musical formation, language skills, reading activity, and intellectual games. Two last questions explored if these activities were being carried at present. The total score (from 0 to 34) was obtained by directly adding the single response scores (Bartrés-Faz et al., 2018). This questionnaire has been previously validated in the Spanish population (Rami et al., 2011). The complete questionnaire can be seen in Supplementary Material.

##### Psychosocial Factors

Sleep quality was assessed with The Jenkins Sleep Evaluation Questionnaire (JSEQ) (Jenkins et al., 1988). Each item was rated on a 6-point Likert scale, where 1 indicated no sleep problems and 6 indicated frequent sleep problems. This questionnaire scored from 4 to 24, where higher scores reflect low sleep quality. The published Spanish translation has also shown good psychometric properties in a validation study (Pallarés-Sanmartín et al., 2019).

To assess the quality of life, we used WHO-QoL-AGE, a 13-item questionnaire scored on a 5-point Likert scale. This questionnaire attempts to assess satisfaction with one's life, living place, general health, i.e., hearing, vision, daily activities, personal relationships, goal achievements, or economic status (The Whoqol Group, 1998). The published Spanish translation has good psychometric data in a validation study (Lucas-Carrasco, 2012).

We used PROMIS^®^ Cognitive Abilities and Cognitive Concerns (Fieo et al., 2016) scales to assess self-perceived cognitive impairment. This scale consisted of 12-item, extracted from the PROMIS^®^ item bank, measuring self-reported cognitive deficits in memory (e.g., my memory is as good as usual), working memory (WM, e.g., I had trouble remembering what I was doing if I was interrupted), attention (e.g., I have had to work hard to pay attention and not make mistakes), processing speed (e.g., my thinking has been slower), or cognitive flexibility. Each item asked participants to answer “within the last 7 days” using five response options: never, rarely (once), sometimes (two or three times), often (about once a day), very often (several times a day).

#### In-person Neuropsychological Assessment

Neuropsychological in-person testing was administered in a single session. Cognitive tests were administered to all subjects in the following fixed order: direct and inverse digit span (Peña-Casanova et al., 2012), Trail Making Test part A and B (TMT-A and TMT-B, respectively; Peña-Casanova et al., 2012), Reasoning Matrix subtest from Wechsler Adult Intelligence Scale- Fourth Edition (WAIS-IV; Wechsler, 2012), Rey Auditory-Verbal Learning Test (RAVLT; Bowler, 2013); Block Design subtest from WAIS-IV (Wechsler, 2012). Letter-Number Sequencing (Peña-Casanova et al., 2012), Digit-Symbol Substitution Test and Cancellation subtests from WAIS-IV, and Corsi block-tapping test (Peña-Casanova et al., 2012). The cognitive assessment session was conducted by two expert neuropsychologists (CPG and VAS) and lasted ~90 min (for details refer to Cattaneo et al., 2018).

#### Data and Statistical Analysis

Differences in sociodemographic status, drug use, and pain-related comorbidities characteristics between participants affected by CP and pain-free participants were explored using one-way ANOVAs and chi-square statistics.

Within people affected by CP we used descriptive statistics to characterize pain location, the number of pain sites, and average intensity over the last week, and pain onset.

Then, we explored group differences between people affected by CP and healthy controls in psychosocial factors, which were sleep and quality of life (The Jenkins Sleep Evaluation Questionnaire and the WHO-QoL-AGE), self-perceived cognitive impairment (PROMIS Cognitive abilities and cognitive concerns scale), CR (CR questionnaire), MH (PHQ-4), and cognitive performance (measured by in-person neuropsychological assessment) running one-way ANOVAs, correcting for age, educational level, and BMI. In these analyses, the variable “Group” (CP, no pain) was used as between the factor of subjects, while psychosocial aspects, such as self-perceived cognitive impairment, CR, MH, and cognitive performance, were used as dependent variables.

In the analyses, to compare CP with healthy controls in cognitive performance, we used scores normalized for age and educational level were possible (Peña-Casanova et al., 2012; Wechsler, 2012), and raw scores when normative data were not available. Statistical tests were performed separately for each test and, to explore the possible influence of MH affliction in group differences in cognition we re-ran significant tests using MH as a covariate.

Within the CP group, the relationship between MH and pain was assessed with multiple regression models. We used MH as a dependent variable, demographic data (age and gender), and CP characteristics (pain intensity in the last week, duration, and the number of pain sites).

In a second step, we added CR to explore its contribution to CP and MH relationship, correcting for possible confounders like sleep quality and BMI.

Finally, to study the role of CR in modulating the relation between pain and MH, we divided CP participants into two pain intensity (higher pain intensity/lower pain intensity) and two CR groups (higher CR/lower CR), based on a percentile 50 of the distribution. Finally, we ran an ANOVA using the variable MH as the dependent variable, and the created groups as between-subject factors, correcting for possible sociodemographic confounders (age, gender, and BMI) and sleep quality.

Statistical analyses were performed using SPSS version 20 (Statistical Package for Social Sciences, Chicago, IL, USA).

## Results

### Differences Between CP and No Pain Groups

#### Sociodemographic Characteristics of the Participants

Sociodemographic data of participants are reported in Table 1.

**Table 1:** Sociodemographic data of participants.

	**No pain**	**CP**	
	***N* = 260**	***N* = 217**	
	**Mean (SD)**	**Mean (SD)**	***p*-value**
Age (Years)	52.5 (6.8)	53.7 (6.7)	0.045^*^
Education (Years)	17.3 (3.5)	16.8 (3.9)	0.145
	**Frequency** **(%)**	**Frequency** **(%)**	***p-*** **value**
Gender (Women)	44.2	51.6	0.108
Professional status			0.304
Unemployed	3.8	6.0	
Retired	5.4	6.9	
Part-time contract	8.8	6.9	
Full-time contract	81.9	80.2	
Professional category			0.059
Unskilled manual labor	3.8	3.7	
Qualified manual labor	26.2	32.3	
Professional (with university studies)	27.3	31.3	
Manager or director (with university studies)	42.7	32.7	
Civil status			0.209
Single	60.0	17.5	
Separated/Divorced	15.4	15.7	
Married	22.7	65.4	
Widower	1.9	1.4	
Monthly incomes			0.613
<1,000€	3.5	3.7	
Between 1,000 and 2,000 €	18.1	15.2	
Between 2,000 and 5,000 €	54.2	62.7	
More than 5,000 €	24.2	18.4	
Medication			<0.001^**^
None	65.0	47.5	
Analgesics (NSAIDs, aspirine…)	2.3	6.5	
Antimigraine	0.4	1.4	
Other drugs (no pain-related)	31.2	34.6	
Mixed medications (no pain and pain)	1.2	10.1	
BMI			<0.001^**^
Underweight	1.5	0.5	
Normal	60.8	46.1	
Overweight	29.6	39.2	
Obese	8.1	14.3	
	**Mean (SD)**	**Mean (SD)**	***p-*** **value**
Audit	2.6 (1.5)	2.9 (2.4)	0.103
	**Frequency** **(%)**	**Frequency** **(%)**	***p-*** **value**
Cannabis			0.221
No	98.5	96.8	
Yes	1.5	3.2	
Nicotine			0.968
No	85.4	85.3	
Yes	14.6	14.7	
Comorbidities			
Hypertension	12.3	16.1	0.237
Cardiovascular diseases	5.8	5.5	0.911
Cholesterol	20.4	25.3	0.201
Diabetes	4.2	4.6	0.842
Arthritis	2.3	3.2	0.541
Thyroid disease	8.5	12.4	0.161
Prothesis	1.5	2.3	0.541
Gastric ulcer	2.3	4.6	0.178

*Post-hoc comparisons revealed that differences were statistically significant between participants without pain and those with CP (p = 0.02)*;

** *p < 0.05*;

* *p < 0.01*.

*SD, standard deviation; NSAIDs, Non-steroidal anti-inflammatory drugs; BMI, body mass index; AUDIT, Alcohol Use Disorders Identification Test*.

Among the 477 individuals included in this study (47.60% women; mean age 53.5 ± 6.7 years), 217 individuals referred pain for more than 3 months (51.6% women, mean age 53.7 ± 6.7 years) and 260 individuals reported no pain (44.2% women, mean age 52.5 ± 6.8 years). There was a significant difference in age between groups. Professional category, professional status and marital status, monthly incomes, and educational level were not different between groups.

Participants with CP had significantly higher BMI compared with people without pain. Moreover, there was a high use of drugs not related to pain in participants with CP. No differences were found between groups on alcohol, nicotine, cannabis use, or other comorbidities.

#### Online Questionnaires: Mental Health, Cognitive Reserve, and Psychosocial Factors

**Figure 2:**
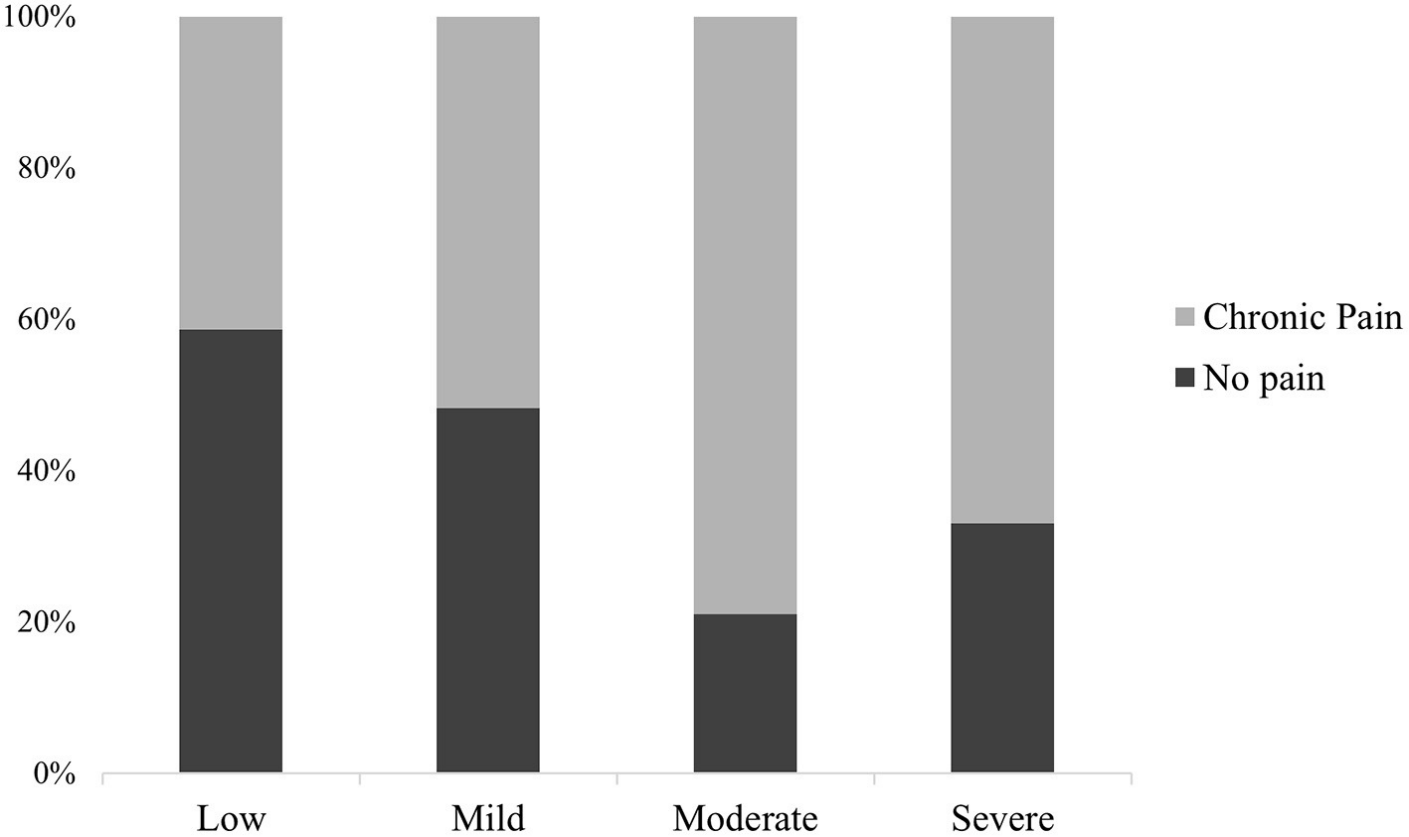
Descriptive distribution of participants with and without CP according to their distress level: low (0–2), mild (3–5), moderate (6–8), and severe (9–12).

People affected by CP presented a higher number of emotional complaints (PHQ-4) (*M* = 2.3, *SD* = 2.2; *p* < 0.001) compared with the no pain group (*M* = 1.4, *SD* = 1.7). Using the psychological distress level diagnosis guideline suggested by Kroenke et al. (2009), 65.9% of CP participants experienced a normal level of distress, 26.3% reported mild distress, 6.9% had moderate distress, and severe distress was present in 0.9% of our pain sample (Table 2; Figure 2).

**Table 2:** Mental health, cognitive reserve and psychosocial factors in no pain and chronic pain group.

	**No pain**	**Chronic pain**	
	**Freq. (%)**	**Freq. (%)**	***p-*value**
Mental health (PHQ-4) (levels)			0.004^*^
Low	77.7	65.9	
Mild	20.4	26.3	
Moderate	1.5	6.9	
Severe	0.4	0.9	
	**Mean (SD)**	**Mean (SD)**	***p-*** **value**
Mental health (PHQ-4) (mean)	1.4 (1.7)	2.3 (2.2)	<0.001^**^
Cognitive reserve	24.8 (4.8)	24.1 (4.9)	0.121
Psychosocial factors			
Self-perceived cognitive impairment (PROMIS)	52.9 (7)	49.1 (9.5)	<0.001^**^
Sleep (JSEQ)	7.1 (3.1)	9 (4.2)	<0.001^**^
Quality of Life (WHOQoL-AGE)	31.5 (6.5)	28.7 (6.4)	<0.001^**^

*Post-hoc comparisons revealed that differences were statistically significant between participants without pain and those with CP (p = 0.02)*;

** *p < 0.05*;

* *p < 0.01*.

*SD, standard deviation; PHQ-4, Patient Health questionnaire; JSEQ, Jenkins Sleep Evaluation Questionnaire*.

The cognitive reserve score was not significantly different between pain groups (*p* = 0.121). However, an increased level of subjective cognitive impairment in the PROMIS^®^ Cognitive Abilities and Cognitive Concerns survey was observed in the CP group (*p* < 0.001).

People affected by CP reported poorer sleep quality than the no pain group (*p* < 0.001) and lower quality of life (*p* < 0.001).

#### In-person Assessment: Cognitive Performance

Results of cognitive tests are summarized in Table 3. All tests were interpreted as mean values in normalized scores, except for the results of the Rey Auditory Verbal Learning Test and Trail Making Test B-A that did not have Spanish normative values. There were no statistical differences between groups, except for the digit span backward (*p* = 0.032), a WM test, which was worst performed by participants with CP.

**Table 3:** Mean normalized and raw scores in neuropsychological assessment.

**Cognitive test**	**Score**	**No CP (mean)**	**CP (mean)**	***p*-value**
WAIS-IV Matrices	Normalized	13.2 (2.6)	13.2 (2.5)	0.981
Block design	Normalized	12.2 (2.9)	11.9 (3.1)	0.414
Trail making Test A	Normalized	11.5 (2.6)	11.3 (2.8)	0.580
Trail making Test B	Normalized	8.9 (2.1)	8.6 (2.1)	0.083
Trail making test B-A	Raw	48.9 (22.4)	51.2 (23.7)	0.698
Digit symbol substitution	Normalized	14 (2.6)	14.7 (2.4)	0.873
Cancellation WAIS-IV	Normalized	11.3 (2.7)	11.6 (2.8)	0.241
Letter-number sequencing	Normalized	14.5 (2.4)	13.9 (2.5)	0.320
Digit span backward	Normalized	11.1 (2.6)	11.6 (2.7)	0.032^*^
Corsi cubes	Normalized	14 (2.5)	14.2 (2.5)	0.269
Digit span forward	Normalized	10.9 (2.5)	11.1 (2.7)	0.529
RAVLT immediat recall	Raw	51.9 (8.8)	52.3 (8.4)	0.117
RAVLT delayed recall	Raw	11.4 (2.8)	11.4 (2.5)	0.324

*Mean normalized scores corrected for age and education, except for RAVLT and Trail Making test B-A (raw scores)*.

* *p < 0.05*.

To explore the possible influence of MH in cognitive functioning differences between groups, we ran a one-way ANOVA using the result of the digit span backward as the dependent variable and the variable “group” (pain, no pain) as between-subject factors. The difference between groups was still significant, and the marginal significant effect of MH on cognition (*F* = 3.085, *p* = 0.080), suggests an independent role of pain in WM (*F* = 6.142, *p* = 0.014).

### Differences Within CP Group

Chronic pain was more frequently located at the back (36.9%), followed by the lower limbs (29.5%) and upper limbs (19.8%), head/face (6.5%), widespread (4.6%), and visceral/pelvic (2.8%) (Figure 2).

The average pain intensity was 3.75 (*SD* = 1.8) on a scale from 0 to 10. From all the subjects who reported having CP pain, only one person rated 0/10 of pain intensity during the last week.

**Figure 3:**
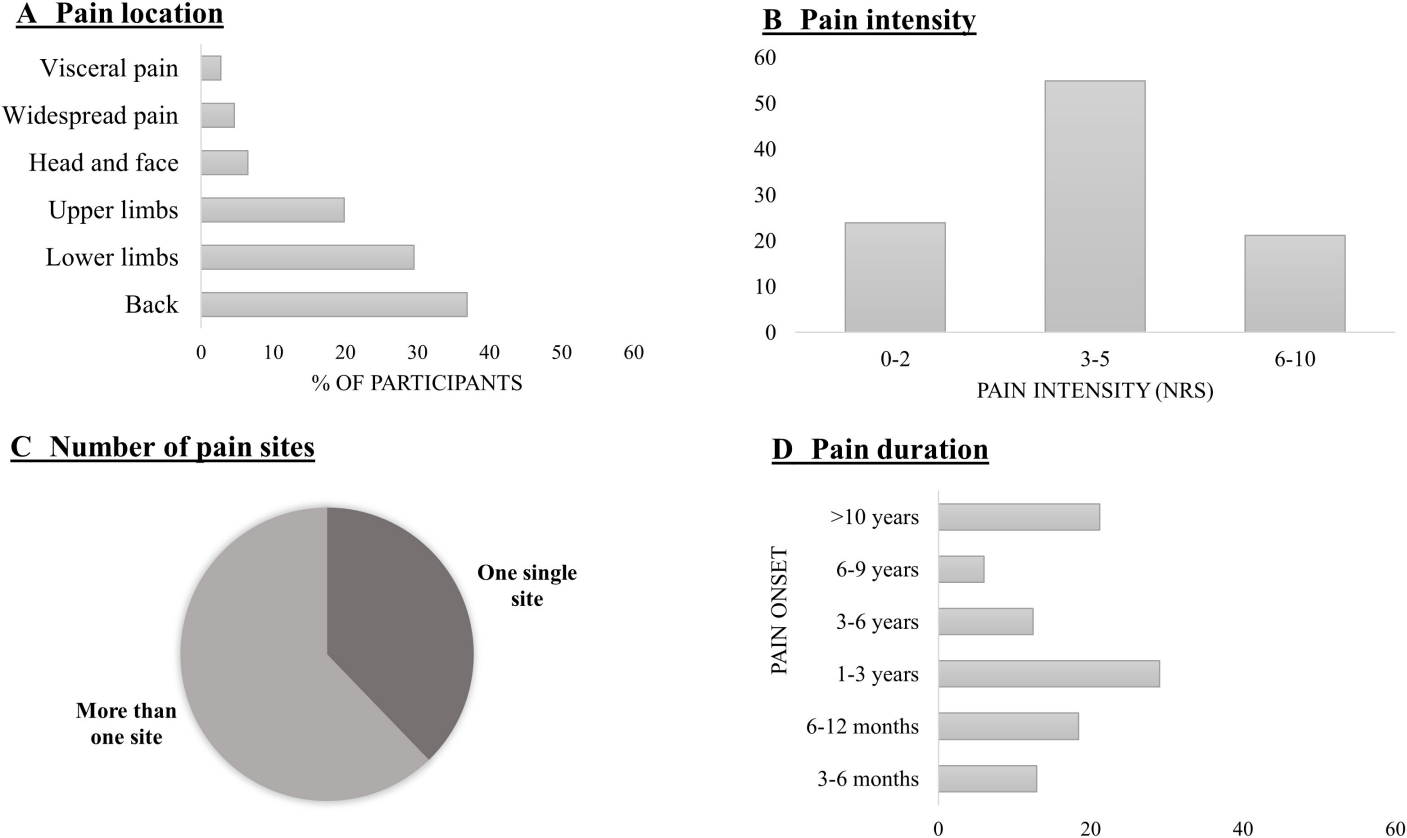
**(A)** Number of participants (in %) in each pain location; **(B)** Percentage of participants according to their last week pain intensity perception in an NRS (Numerical Rating Scale). Ratings have been classified into three main groups: from 0 to 2; 3–5; 6–10; **(C)** Population (in %) with one pain site (or one kind of pain) and more than one site; **(D)** Participants' pain onset, classified in 3–6 months; 6–12 months; 1–3 years; 3–6 years; 6–9 years, and more than 10 years.

From our sample, 37.8% had a single site pain, while 62.2% reported two or more pain sites. A relatively small proportion of participants experienced pain for the last 3–6 (12.9%) and 6–12 months (18.4%), while most participants suffered from CP for more than 12 months (68.7%) (Figure 3).

#### Pain Intensity, Cognitive Reserve Interaction, and Mental Health in CP

Multiple regression in CP sample analysis revealed that age (β = −0.212; *p* = 0.001) and pain intensity (β = 0.167, *p* = 0.016) were significantly associated with MH. No significant associations were found for gender (β = 0.008; *p* = 907), pain duration (β = 0.079, *p* = 0.238), or the number of pain sites (β = 0.102, *p* = 0.138).

When we included into the regression model CR and psychosocial variables, we found that MH was still significantly associated with age (β = −0.173, *p* = 0.005) and pain intensity (β = 0.131, *p* = 0.048), but in addition, it resulted related to sleep quality (β = 0.332, *p* < 0.001) and CR (β = − 0.260, *p* < 0.001) (as shown in Table 4).

**Table 4:** Multiple regressions: the influence of chronic pain and psychosocial factors on mental health.

**Domain**	**β**	***p*-value**
Age	−0.173	0.005^**^
Gender	0.008	0.895
Pain duration	0.062	0.308
Pain intensity	0.131	0.048^*^
Multisite pain	0.041	0.525
BMI	−0.041	0.523
Sleep	0.332	<0.001^**^
CR	−0.260	<0.001^**^

** *p < 0.05*;

* *p < 0.01. Table reports β-standardized scores and p-values*.

*BMI, body mass index; CR, Cognitive Reserve*.

**Figure 4:**
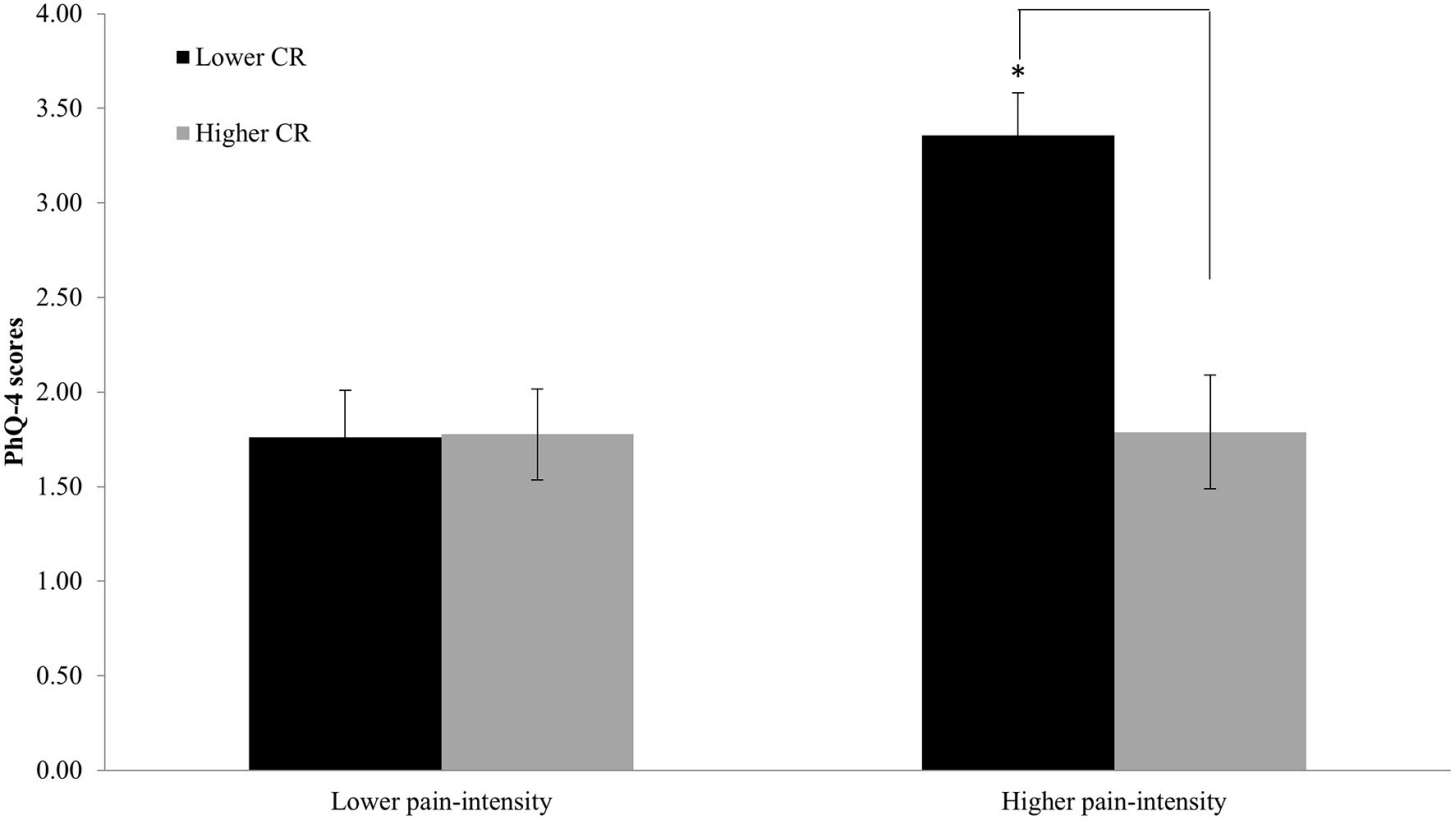
Distribution of CP participants (Higher pain intensity/Lower pain intensity; Higher CR/Lower CR) according to the percentile 50. Participants with higher CR showed less impact on mental health in the higher pain-intensity group. *p < 0.05.

The evaluation of the interaction between CR and pain intensity using pain intensity groups (higher, lower) and CR groups (higher, lower) as between-subject factors, revealed an intensity main effect (*F* = 8.639; *p* = 0.004), indicating worse MH in people with higher pain intensity. The CR main effect (*F* = 5.767; *p* = 0.017) indicated that people with high CR showed lower MH affliction. Crucially, we found a significant pain intensity and reserve interaction (*F* = 6.956; *p* = 0.009; Figure 4).

## Discussion

The current study explored the impact of CP on psychosocial factors, MH, and cognition. Furthermore, we aimed to examine the role of CR in the relationship between MH and CP clinical characteristics in middle-aged adults.

Our main results suggested that: (1) CP participants showed worst levels of WM and MH, and (2) the presence of high/low CR modifies the interaction between pain intensity and MH.

We found a high prevalence of CP (45.5%, 217 participants) in our cohort, in line with previous reports of a peak prevalence in late middle-aged adults (50–65 years), affecting up to 20–80% of people (Gibson and Lussier, 2012). CP at multiple pain sites was the most prevalent pain condition (62.2%; Brady et al., 2015; Suvinen et al., 2016; Finney et al., 2017), CP in the back and joints in the lower limbs were the most prevalent pain sites (Ligthart et al., 2014; Maixner et al., 2016), and 40% of people affected by CP referred the presence of pain for more than 3 years.

Our CP group showed a higher presence of overweight compared with people without pain. There may be a bidirectional causality, wherein CP may result in weight gain by reducing physical activity (Verbunt et al., 2003), and individuals could have structural body changes causing pain (Lake et al., 2000). The fact that the CP group consumed significantly more medication, which is associated with higher BMI, suggested that this group has worse general health. Finally, overweight has been related previously with other domains referred to in our study, as well as sleep quality (Hargens et al., 2013) or cognitive impairment (Nilsson and Nilsson, 2009).

Our findings were consistent with previous research, reporting an association between CP with sleep, mood, and psychosocial alterations (Edwards et al., 2016). Pain and sleep have a reciprocal, interdependent relationship, wherein poor sleep can predict pain in certain conditions and compounds the pain experience (Turk et al., 2016). Furthermore, sleep deprivation can lower the pain threshold, affecting the cognitive ability to cope with pain, increasing ratings of pain intensity, which in turn exacerbates poor sleep (Okifuji and Hare, 2011).

A further finding of our study prolonged experience of pain was associated with significantly lower quality of life, cognitive and mood consequences, such as mild depressive symptoms (PHQ-4), and self-perceived cognitive impairment (PROMIS). Unquestionably, feeling pain may evolve from typical reactive emotional symptoms related to stress (Hammen, 2005) to clinically relevant depression (DeVeaugh-Geiss et al., 2010). This close relationship between emotions and pain is a complex experience that is perceived differently across people and varies even within individuals depending on context (Bushnell et al., 2013). As for other adaptive affective responses, such as fear and anxiety, the level of emotional facilitation of pain when it comes to some individuals can be maladaptive, leading to long-term suffering (Gandhi et al., 2020). Additionally, people with CP reported more subjective cognitive complaints. On one hand, pain acts through the consumption of attentional resources, and, on the other, through psychological and emotional aspects. This can give rise to feelings of discomfort, leading some individuals to overestimate their difficulties, including subjective emotional and cognitive impairment, which can be ultimately transferred in everyday behavior.

Concerning the cognitive performance in our sample, almost all domains were not affected when we compared CP with no pain group. This is coherent with the fact that we are describing a healthy and young sample, and exclusion criteria were specially chosen to avoid other variables that could affect cognition (use of psychiatric or neurologic disorders or opioids). In addition, we also analyzed possible cofounders as age, educational level, or BMI. Notwithstanding, our results indicated that people with CP showed lower WM performance, in the digit span backward test, and this was consistent with several previous studies (Sjøgren et al., 2005; Veldhuijzen et al., 2006; Buhle and Wager, 2010). WM has been related to a better capacity to choose cognitive strategies when facing a situation or task (Hilbert et al., 2015), and it is considered as one of several functions within the executive control domain strongly related to attentional processes (McCabe et al., 2010). Indeed, evidence suggested that selective attention facilitates working memory processing, by preventing inference from unrelated distractions, while maintaining attention to the relevant targets (Legrain et al., 2011). Thus, painful stimuli or emotional discomfort could act as distractors in our pain participants, causing an abnormal allocation of attentional resources to pain stimuli and consequently impact WM capacity.

At a neurobiological level, two main regions in the frontal cortex, related with both WM and pain processing, could represent the substrate of this relation. The dorsolateral prefrontal cortex (DLPFC) and orbitofrontal cortex were evidently both involved in the ability to cope with pain and to inhibit pain, respectively (Bunk et al., 2019). Several studies not only have shown decreased gray matter in left DLPFC and orbitofrontal cortex in CP patient but also how these abnormalities can be reversed with CP resolution (Davis and Moayedi, 2013; Seminowicz and Moayedi, 2017; Ong et al., 2019). Furthermore, an association between these areas and attentional processing and WM has been demonstrated (Davis et al., 2008). For instance, patients with back pain, fibromyalgia, or CRPS have shown deficits in emotional decision-making when they were tested with the Iowa gambling task (Apkarian et al., 2004; Tamburin et al., 2014). Thus, the anatomical changes observed in patients with CP may well be an underlying factor in their altered performance in cognitive tasks. It is important to point out that when we explored if the cognitive function was primarily due to pain or to MH results showed that, although MH had a partial contribution, the pain has an independent effect in WM, as previously seen by Bunk et al. in their review (Bunk et al., 2019).

In this sense, it has been also demonstrated that persistent and CP is associated with alteration in the activity of the Default Mode Network (DMN) and the connectivity between its nodes (medial prefrontal cortex, lateral parietal cortex/angular gyrus, and posterior cingulate cortex/precuneus) (Baliki et al., 2008; Tessitore et al., 2013), consistently related with WM dysfunctions (Mazoyer et al., 2001; Sambataro et al., 2010), coping strategies (Santarnecchi et al., 2018) and several MH pathological conditions (refer to Broyd et al., 2009 for a review) (Broyd et al., 2009). Recent evidence from non-invasive brain stimulation (NIBS) indicates that stimulation of nodes of the DMN is associated with WM performance in healthy adults (Pupíková et al., 2021) or could be a promising therapeutic approach to reduce cognitive and mood disorders in different pathologies (Alexoudi et al., 2019, 2020; Chou et al., 2020). Finally, it has been recently shown that transcranial direct current stimulation (tDCS) modulates pain perception, but depending on the age, the brain target should be M1 (for older adults) or DLPFC (for younger adults), although both sides increased WM ability (Saldanha et al., 2020). These gains are related to different connections of these structures with pain processing, but it has also an impact on cognitive and emotional features related to pain perception.

What we considered as one of the most relevant findings was that the relationship between MH and pain intensity was modulated by CR, wherein only in the high pain intensity group, participants with higher CR showed less MH affliction, compared with lower CR participants. Under certain assumptions, this could be interpreted as individuals with higher levels of CR can handle and manage pain and attentional resources more efficiently (with lower suffering) than people with low CR.

In this line previous studies have seen a CR moderator effect between mood and cognition (Pertl et al., 2017; Opdebeeck et al., 2018; Huang et al., 2019), wherein higher CR may increase the ability to manage difficulties, employing the best strategy for performing a task and better allocating cognitive resources (Barulli et al., 2013). This then allows some individuals to be less prone to attend to pain or depressive symptoms and more capable to easily “tune out” it.

Cognitive reserve did not prevent the pre-determinate biological factors from spurring the pathology, as it acted as a complementary mechanism, which modified how the brain responds to that factors once they were present (Barulli and Stern, 2013, 2018). In this sense, it is important to note that the CR levels of our sample were similar in both groups. Once pathology was present, as said before, CR could be associated with the ability of an individual to resolve difficulties and use optimally cognitive resources and alternative processes and strategies (Barulli et al., 2013).

This role of CR in modulating the relation between CP and MH, *via* better allocation of cognitive and attentional resources, could potentially be extended to other consequences of CP. In this line, one can speculate a more general model of the moderator role of CR in CP, considering that CR could exert, through the same mechanisms, a similar protective effect also for the development of pain-related comorbidities or cognitive dysfunctions like the WM impairment we found in our sample.

In this sense, since pain is also actively determined by expectations and learned experiences, more general-domain functional descending top-down modulatory effects orchestrated by prefrontal structures linked with CR may buff the ability to manage the bottom-up and involuntary capture of attention by pain (Legrain et al., 2009a,b; Wiech, 2016), affecting the processing of nociceptive stimuli at early levels by biasing somatosensory brain activity (Legrain et al., 2002). From a pathophysiological point of view, the study of Villemure and Schweinhardt (2010) suggested that supraspinal pain processing, and specifically, increased activity in the lateral orbitofrontal cortex [closely related to CR; refer to Gomez-Beldarrain et al. (2016)] induced by negative emotions, could lead to descending facilitatory pathways by increasing nociceptive signals transmitted from the spinal cord to the brain.

Regarding descending modulation networks in fMRI studies (also known as antinociceptive system) in fMRI studies, Kucyi et al. (2013) and Kucyi and Davis (2015) introduced the concept of a dynamic “pain connectome,” in which differences in mind wandering from pain and attention to pain stimuli are associated with cognitive performance and brain functioning. Increased activations of the above-mentioned DMN, were seen when the minds of people wander away from pain. Moreover, strong interactions between the DMN and periaqueductal gray, an opiate-rich region mediating pain suppression (Pessoa, 2008), resulting in weaker anatomical connections and the antinociceptive system were found in subjects with difficulty tuning out pain.

This tuning out pain difficulty is often explained in terms of the specific construct of a “hyper-vigilance” to pain, i.e., a tendency to increase attentional allocation to pain-related information, related to habits to attend to bodily sensations or the persistent search for pain relief in patients (Crombez et al., 2005). Individuals who expect somatosensory stimuli to be painful (fear and catastrophic thoughts) show more attentional interruption by non-painful somatosensory stimuli that are delivered at the same location (Crombez et al., 2005) and this is associated with greater activity in operculo-insular and mid-cingulate cortex (Seminowicz and Davis, 2006). Further, individuals with constitutively limited attentional resources or cognitive flexibility are at greater risk of more difficulties to cope with pain, partly due to a decision-making ability or may be linked to the mechanisms of pain chronicity (Attal et al., 2014).

From this perspective, the construct of CR could be used in the prevention or treatment of CP, as it has already been seen in Alzheimer's disease (Scarmeas and Stern, 2003; Arenaza-Urquijo et al., 2015). CP approaches based on cognition have been showing to be effective. As Kucyi et al. (2016) stated in their study, CP treatment should be addressed to change the cognitive context in which pain occurs, maladaptive neuroplastic changes occurring in response to spontaneous neuronal pain-related activity should be modified. In their study about cognitive-behavioral therapy, they found changes in fMRI, like increased intrinsic connectivity between the ventrolateral prefrontal cortex and DMN (Kucyi et al., 2016). Cognitive training has also been shown to be effective in improving cognitive skills and decrease pain intensity (Baker et al., 2018). In the absence of an effective pharmacological treatment for CP, we can consider that the potential role of lifestyles factors (for example, training cognitive skills in daily life), understanding the neural mechanisms underlying expectations, emotional state, or focus attention on pain, seems crucial.

### Limitations

The main limitation of this study was that our sample was non-homogenous, with a high prevalence of individuals with high education, which may lead to results that may not be representative of the general population. Moreover, the sample of the study is relatively small, and most measures are self-reported. Another limitation was that our participants were healthy, that is, not patients in a pain unit, and they did not have high levels of pain in intensity or pain-related impairments (cognition, pain interference, or affective disorders). Future research should consider the potential effects of cognition and emotion in pain modulation more carefully, for example focusing on pain catastrophizing and coping strategies, their relationship with CR, and their influence on pain.

Finally, this study has some disadvantages derived from the cross-sectional design, where the principal flaw is that we cannot determine if the relationship explained above between pain and mental health is cause or effect.

## Conclusions

In summary, CR may play a determinant protective role in CP, reducing its impact on psychological and MH well-being, and buff endogenous control mechanisms. Persistent pain can be detrimental to the brain, decreasing the ability of an individual to endogenously control it and lead to many comorbidities, concluding with the idea that “no pain, healthy brain.”

Understanding the dynamic relationship between CP, MH, and CR, and that higher CR in healthy adults was associated with better emotional decision-making and strategies selection to cope with pain and MH, resulting in relevant implications for developing new spots on CP and treatment approaches.

This provided a good starting point for further research. Longitudinal assessments with appropriate control conditions are needed to determine how CR and changes in pain connectome dynamics mediate the effects in middle-aged adults with CP.

## Data Availability Statement

The raw data supporting the conclusions of this article will be made available by the authors, without undue reservation.

## Ethics Statement

The studies involving human participants were reviewed and approved by Comité d'Ètica i Investigació Clínica de la Unió Catalana d'Hospitals. The patients/participants provided their written informed consent to participate in this study.

## Author Contributions

MDS, GC, SA, VA-S, CP-G, JS-S, AP-L, DB-F, JT, and SD-G had made substantial contributions to conception, design, and interpretation of data. AP-L, DB-F, JT, SA, VA-S, CP-G, and JS-S contributed to revising it critically for important intellectual content. MDS, GC, and SD-G participated in drafting the manuscript, made substantial contributions to analysis, and interpretation of data. SA, VA-S, CP-G, JS-S, and SD-G had made substantial contribution to acquisition of data. All authors have given final approval of the version to be submitted.

## Funding

MDS was supported by ERANET fund from the Instituto de Salud Carlos III of Spain. AP-L is a co-founder of Linus Health and TI Solutions AG, serves on the scientific advisory boards for Starlab Neuroscience, Neuroelectrics, Magstim Inc., and MedRhythms, and is listed as an inventor on several issued and pending patents on the real-time integration of non-invasive brain stimulation with electroencephalography and magnetic resonance imaging. DB-F was funded by a Spanish Ministry of Science, Innovation and Universities (MICIU/FEDER; RTI2018-095181-B-C21) and an ICREA Academia 2019 research grants, and also supported by an ICREA Academia 2019 grand award, JT was partly supported Fundació Joan Ribas (Araquistain_FJRA), AGAUR, Agència de Gestió d'Ajuts Universitaris i de Recerca. Convocatòria 2018 d'Indústria del Coneixement (modalitat PRODUCTE) and FEDER, Fons Europeu de Desenvolupament Regional (2018 PROD 00172), Fundació La Marató De TV3 (201735.10), and European Commission - H2020/Call H2020-SC1-2016-2017 (RIA) (Grant Agreement No. 777107).

## Conflict of Interest

AP-L received funding from MagStim Inc. The funder was not involved in the study design, collection, analysis, interpretation of data, the writing of this article or the decision to submit it for publication. The remaining authors declare that the research was conducted in the absence of any commercial or financial relationships that could be construed as a potential conflict of interest.

## Publisher's Note

All claims expressed in this article are solely those of the authors and do not necessarily represent those of their affiliated organizations, or those of the publisher, the editors and the reviewers. Any product that may be evaluated in this article, or claim that may be made by its manufacturer, is not guaranteed or endorsed by the publisher.

## References

[B1] Alexoudi A. Patrikelis P. Deftereos S. Fasilis T. Karakalos D. Verentzioti A.. (2019). Effects of anodal transcranial direct current stimulation on cognitive dysfunction in patients with progressive supranuclear palsy. Psychiatriki 30, 320–328. 10.22365/jpsych.2019.304.320 32283535

[B2] Alexoudi A. Patrikelis P. Fasilis T. Deftereos S. Sakas D. Gatzonis S. (2020). Effects of anodal tDCS on motor and cognitive function in a patient with multiple system atrophy. Disabil. Rehabil. 42, 887–891. 10.1080/09638288.2018.1510043 30345833

[B3] Apkarian A. V. Baliki M. N. Farmer M. A. (2013). Predicting transition to chronic pain. Curr. Opin. Neurol. 26, 360–367. 10.1097/WCO.0b013e32836336ad 23823463 PMC4887742

[B4] Apkarian A. V. Sosa Y. Krauss B. R. Thomas P. S. Fredrickson B. E. Levy R. E.. (2004). Chronic pain patients are impaired on an emotional decision-making task. Pain 108, 129–136. 10.1016/j.pain.2003.12.015 15109516

[B5] Arenaza-Urquijo E. M. Wirth M. Chételat G. (2015). Cognitive reserve and lifestyle: moving towards preclinical Alzheimer's disease. Front. Aging Neurosci. 7:134. 10.3389/fnagi.2015.00134 26321944 PMC4530312

[B6] Attal N. Masselin-Dubois A. Martinez V. Jayr C. Albi A. Fermanian J.. (2014). Does cognitive functioning predict chronic pain? Results from a prospective surgical cohort. Brain 137, 904–917. 10.1093/brain/awt354 24441173

[B7] Badia X. Muriel C. Gracia A. Manuel Núñez-Olarte J. Perulero N. Gálvez R.. (2003). Validación española del cuestionario Brief Pain Inventory en pacientes con dolor de causa neoplásica. Med. Clín. 120, 52–59. 10.1016/S0025-7753(03)73601-X 12570914

[B8] Baker K. S. Georgiou-Karistianis N. Lampit A. Valenzuela M. Gibson S. J. Giummarra M. J. (2018). Computerised training improves cognitive performance in chronic pain: a participant-blinded randomised active-controlled trial with remote supervision. Pain 159, 644–655. 10.1097/j.pain.0000000000001150 29447133

[B9] Baker T. A. Krok-Schoen J. L. O'Connor M. L. Brooks A. K. (2016). The influence of pain severity and interference on satisfaction with pain management among middle-aged and older adults. Pain Res. Manag. 2016:9561024. 10.1155/2016/9561024 28100956 PMC5215497

[B10] Baliki M. N. Apkarian A. V. (2015). Nociception, pain, negative moods, and behavior selection. Neuron 87, 474–491. 10.1016/j.neuron.2015.06.005 26247858 PMC4529956

[B11] Baliki M. N. Geha P. Y. Apkarian A. V. Chialvo D. R. (2008). Beyond feeling: chronic pain hurts the brain, disrupting the default-mode network dynamics. J. Neurosci. 28, 1398–1403. 10.1523/JNEUROSCI.4123-07.2008 18256259 PMC6671589

[B12] Bartrés-Faz D. Cattaneo G. Solana J. Tormos J. M. Pascual-Leone A. (2018). Meaning in life: resilience beyond reserve. Alzheimers Res. Ther. 10:47. 10.1186/s13195-018-0381-z 29793549 PMC5968537

[B13] Barulli D. Stern Y. (2013). Efficiency, capacity, compensation, maintenance, plasticity: emerging concepts in cognitive reserve. Trends Cogn. Sci. 17, 502–509. 10.1016/j.tics.2013.08.012 24018144 PMC3840716

[B14] Barulli D. Stern Y. (2018). “Cognitive reserve: theory, measurement, and evidence,” in APA Handbook of Dementia APA Handbooks in Psychology^®^, eds G. E. Smith and S. T. Farias (Washington, DC: American Psychological Association), 357–368.

[B15] Barulli D. J. Rakitin B. C. Lemaire P. Stern Y. (2013). The influence of cognitive reserve on strategy selection in normal aging. J. Int. Neuropsychol. Soc. 19, 841–844. 10.1017/S1355617713000593 23714237 PMC3755348

[B16] Bowler D. (2013). “Rey auditory verbal learning test (rey AVLT),” in Encyclopedia of Autism Spectrum Disorders, ed. Volkmar F. R. (New York, NY: Springer), 2591–2595.

[B17] Brady S. R. E. Mamuaya B. B. Cicuttini F. Wluka A. E. Wang Y. Hussain S. M.. (2015). Body composition is associated with multisite lower body musculoskeletal pain in a community-based study. J. Pain 16, 700–706. 10.1016/j.jpain.2015.04.006 25958316

[B18] Broyd S. J. Demanuele C. Debener S. Helps S. K. James C. J. Sonuga-Barke E. J. S. (2009). Default-mode brain dysfunction in mental disorders: a systematic review. Neurosci. Biobehav. Rev. 33, 279–296. 10.1016/j.neubiorev.2008.09.002 18824195

[B19] Brucki S. M. Brucki S. M. (2016). Chronic pain and cognition. Arquivos Neuro-Psiquiatr. 74, 359–360. 10.1590/0004-282X20160062 27191229

[B20] Buhle J. Wager T. D. (2010). Performance-dependent inhibition of pain by an executive working memory task. Pain 149, 19–26. 10.1016/j.pain.2009.10.027 20129735 PMC4229048

[B21] Bunk S. Preis L. Zuidema S. Lautenbacher S. Kunz M. (2019). Executive functions and pain A systematic review. Zeitschrift Neuropsychol. 30, 169–196. 10.1024/1016-264X/a000264

[B22] Bushnell M. C. Ceko M. Low L. A. (2013). Cognitive and emotional control of pain and its disruption in chronic pain. Nat. Rev. Neurosci. 14, 502–511. 10.1038/nrn3516 23719569 PMC4465351

[B23] Carretero M. Á. G. Ruiz J. P. N. Delgado J. M. M. González C. O. (2016). Validación del test para la identificación de trastornos por uso de alcohol en población universitaria: AUDIT y AUDIT-C. Adicciones 28, 194–204. 10.20882/adicciones.775 26990260

[B24] Cattaneo G. Bartrés-Faz D. Morris T. P. Sánchez J. S. Macià D. Tarrero C.. (2018). The barcelona brain health initiative: a cohort study to define and promote determinants of brain health. Front. Aging Neurosci. 10:321. 10.3389/fnagi.2018.00321 30405394 PMC6204574

[B25] Cattaneo G. Bartrés-Faz D. Morris T. P. Sánchez J. S. Macià D. Tormos J. M.. (2020). The barcelona brain health initiative: cohort description and first follow-up. PLoS ONE 15:e0228754. 10.1371/journal.pone.0228754 32045448 PMC7012435

[B26] Chou T. Hooley J. M. Camprodon J. A. (2020). Transcranial direct current stimulation of default mode network parietal nodes decreases negative mind-wandering about the past. Cogn. Ther. Res. 44, 10–20. 10.1007/s10608-019-10044-9 33456096 PMC7810264

[B27] Crombez G. Van Damme S. Eccleston C. (2005). Hypervigilance to pain: an experimental and clinical analysis. Pain 116, 4–7. 10.1016/j.pain.2005.03.035 15927387

[B28] Davis K. D. Moayedi M. (2013). Central mechanisms of pain revealed through functional and structural MRI. J. Neuroimm. Pharmacol. 8, 518–534. 10.1007/s11481-012-9386-8 22825710

[B29] Davis K. D. Pope G. Chen J. Kwan C. L. Crawley A. P. Diamant N. E. (2008). Cortical thinning in ibs: implications for homeostatic, attention, and pain processing. Neurology 70, 153–154. 10.1212/01.wnl.0000295509.30630.10 17959767

[B30] DeVeaugh-Geiss A. M. West S. L. Miller W. C. Sleath B. Gaynes B. N. Kroenke K. (2010). The adverse effects of comorbid pain on depression outcomes in primary care patients: results from the ARTIST trial. Pain Med. 11, 732–741. 10.1111/j.1526-4637.2010.00830.x 20353408

[B31] Edwards R. R. Dworkin R. H. Sullivan M. D. Turk D. C. Wasan A. D. (2016). The role of psychosocial processes in the development and maintenance of chronic pain. J. Pain 17, T70–92. 10.1016/j.jpain.2016.01.001 27586832 PMC5012303

[B32] Fieo R. Ocepek-Welikson K. Kleinman M. Eimicke J. P. Crane P. K. Cella D.. (2016). Measurement equivalence of the patient reported outcomes measurement information system^®^ (PROMIS^®^) applied cognition - general concerns, short forms in ethnically diverse groups. Psychol. Test Assess. Model. 58, 255–307. Available online at: https://psycnet.apa.org/record/2016-42060-002 28523238 PMC5433382

[B33] Finney A. Dziedzic K. S. Lewis M. Healey E. (2017). Multisite peripheral joint pain: a cross-sectional study of prevalence and impact on general health, quality of life, pain intensity and consultation behaviour. BMC Musculoskelet Disord. 18:535. 10.1186/s12891-017-1896-3 29246141 PMC5732469

[B34] Gandhi W. Rosenek N. R. Harrison R. Salomons T. V. (2020). Functional connectivity of the amygdala is linked to individual differences in emotional pain facilitation. Pain 161, 300–307. 10.1097/j.pain.0000000000001714 31613866

[B35] Generaal E. Vogelzangs N. Penninx B. W. J. H. Dekker J. (2017). Insomnia, sleep duration, depressive symptoms, and the onset of chronic multisite musculoskeletal pain. Sleep 40. 10.1093/sleep/zsw030 28364456

[B36] Gibson S. J. Lussier D. (2012). Prevalence and relevance of pain in older persons. Pain Med. 13, S23–S26. 10.1111/j.1526-4637.2012.01349.x 22497744

[B37] Gómez-Beldarrain M. Anton-Ladislao A. Aguirre-Larracoechea U. Oroz I. García-Moncó J. C. (2015). Low cognitive reserve is associated with chronic migraine with medication overuse and poor quality of life. Cephalalgia 35, 683–691. 10.1177/0333102414553822 25304767

[B38] Gomez-Beldarrain M. Oroz I. Zapirain B. G. Ruanova B. F. Fernandez Y. G. Cabrera A.. (2016). Right fronto-insular white matter tracts link cognitive reserve and pain in migraine patients. J. Head. Pain 17:4. 10.1186/s10194-016-0593-1 26830863 PMC4735096

[B39] Hammen C. (2005). Stress and depression. Annu. Rev. Clin. Psychol. 1, 293–319. 10.1146/annurev.clinpsy.1.102803.143938 17716090

[B40] Hargens T. A. Kaleth A. S. Edwards E. S. Butner K. L. (2013). Association between sleep disorders, obesity, and exercise: a review. Nat. Sci. Sleep 5, 27–35. 10.2147/NSS.S34838 23620691 PMC3630986

[B41] Hilbert S. Nakagawa T. T. Puci P. Zech A. Bühner M. (2015). The digit span backwards task: verbal and visual cognitive strategies in working memory assessment. Eur. J. Psychol. Assess. 31, 174–180. 10.1027/1015-5759/a000223

[B42] Huang C.-M. Fan Y.-T. Lee S.-H. Liu H.-L. Chen Y.-L. Lin C.. (2019). Cognitive reserve-mediated neural modulation of emotional control and regulation in people with late-life depression. Soc. Cogn. Affect. Neurosci. 14, 849–860. 10.1093/scan/nsz054 31603228 PMC6847904

[B43] Jenkins C. D. Stanton B. A. Niemcryk S. J. Rose R. M. (1988). A scale for the estimation of sleep problems in clinical research. J. Clin. Epidemiol. 41, 313–321. 10.1016/0895-4356(88)90138-2 3351539

[B44] Karaman S. Karaman T. Dogru S. Onder Y. Citil R. Bulut Y. E.. (2014). Prevalence of sleep disturbance in chronic pain. Eur. Rev. Med. Pharmacol. Sci. 18, 2475–2481. Available online at: https://www.europeanreview.org/article/7760 25268092

[B45] Kawai K. Kawai A. T. Wollan P. Yawn B. P. (2017). Adverse impacts of chronic pain on health-related quality of life, work productivity, depression and anxiety in a community-based study. Fam. Pract. 34, 656–661. 10.1093/fampra/cmx034 28444208 PMC6260800

[B46] Keller S. Bann C. M. Dodd S. L. Schein J. Mendoza T. R. Cleeland C. S. (2004). Validity of the brief pain inventory for use in documenting the outcomes of patients with noncancer pain. Clin. J. Pain 20, 309–318. 10.1097/00002508-200409000-00005 15322437

[B47] Kocalevent R.-D. Finck C. Jimenez-Leal W. Sautier L. Hinz A. (2014). Standardization of the Colombian version of the PHQ-4 in the general population. BMC Psychiatry 14:205. 10.1186/1471-244X-14-205 25037706 PMC4223637

[B48] Kroenke K. Spitzer R. L. Williams J. B. W. Löwe B. (2009). An ultra-brief screening scale for anxiety and depression: the PHQ-4. Psychosomatics 50, 613–621. 10.1016/S0033-3182(09)70864-3 19996233

[B49] Kucyi A. Davis K. D. (2015). The dynamic pain connectome. Trends Neurosci. 38, 86–95. 10.1016/j.tins.2014.11.006 25541287

[B50] Kucyi A. Salomons T. V. Davis K. D. (2013). Mind wandering away from pain dynamically engages antinociceptive and default mode brain networks. Proc. Natl. Acad. Sci. USA. 110, 18692–18697. 10.1073/pnas.1312902110 24167282 PMC3832014

[B51] Kucyi A. Salomons T. V. Davis K. D. (2016). Cognitive behavioral training reverses the effect of pain exposure on brain network activity. Pain 157, 1895–1904. 10.1097/j.pain.0000000000000592 27101426

[B52] Lake J. K. Power C. Cole T. J. (2000). Back pain and obesity in the 1958 British birth cohort cause or effect? J. Clin. Epidemiol. 53, 245–250. 10.1016/S0895-4356(99)00155-9 10760633

[B53] Landrø N. I. Fors E. A. Våpenstad L. L. Holthe Ø. Stiles T. C. Borchgrevink P. C. (2013). The extent of neurocognitive dysfunction in a multidisciplinary pain centre population. Is there a relation between reported and tested neuropsychological functioning? Pain 154, 972–977. 10.1016/j.pain.2013.01.013 23473784

[B54] Leadley R. M. Armstrong N. Reid K. J. Allen A. Misso K. V. Kleijnen J. (2014). Healthy aging in relation to chronic pain and quality of life in Europe. Pain Pract. 14, 547–558. 10.1111/papr.12125 24138082

[B55] Legrain V. Crombez G. Verhoeven K. Mouraux A. (2011). The role of working memory in the attentional control of pain. Pain 152, 453–459. 10.1016/j.pain.2010.11.024 21238855

[B56] Legrain V. Damme S. V. Eccleston C. Davis K. D. Seminowicz D. A. Crombez G. (2009a). A neurocognitive model of attention to pain: behavioral and neuroimaging evidence. Pain 144, 230–232. 10.1016/j.pain.2009.03.020 19376654

[B57] Legrain V. Guérit J.-M. Bruyer R. Plaghki L. (2002). Attentional modulation of the nociceptive processing into the human brain: selective spatial attention, probability of stimulus occurrence, and target detection effects on laser evoked potentials. Pain 99, 21–39. 10.1016/S0304-3959(02)00051-9 12237181

[B58] Legrain V. Perchet C. García-Larrea L. (2009b). Involuntary orienting of attention to nociceptive events: neural and behavioral signatures. J. Neurophysiol. 102, 2423–2434. 10.1152/jn.00372.2009 19692512

[B59] Ligthart L. Visscher C. M. van Houtem C. M. H. H. Geels L. M. Vink J. M. de Jongh A.. (2014). Comorbidity among multiple pain symptoms and anxious depression in a Dutch population sample. J. Pain 15, 945–955. 10.1016/j.jpain.2014.06.007 24981129

[B60] Lucas-Carrasco R. (2012). The WHO quality of life (WHOQOL) questionnaire: Spanish development and validation studies. Qual. Life Res. 21, 161–165. 10.1007/s11136-011-9926-3 21611868

[B61] Maixner W. Fillingim R. B. Williams D. A. Smith S. B. Slade G. D. (2016). Overlapping chronic pain conditions: implications for diagnosis and classification. J. Pain 17, T93–T107. 10.1016/j.jpain.2016.06.002 27586833 PMC6193199

[B62] Mazoyer B. Zago L. Mellet E. Bricogne S. Etard O. Houdé O.. (2001). Cortical networks for working memory and executive functions sustain the conscious resting state in man. Brain Res. Bull. 54, 287–298. 10.1016/S0361-9230(00)00437-8 11287133

[B63] McCabe D. P. Roediger H. L. McDaniel M. A. Balota D. A. Hambrick D. Z. (2010). The relationship between working memory capacity and executive functioning: evidence for a common executive attention construct. Neuropsychology 24, 222–243. 10.1037/a0017619 20230116 PMC2852635

[B64] Moriarty O. Finn D. P. (2014). Cognition and pain. Curr. Opin. Supp. Palliat. Care 8, 130–136. 10.1097/SPC.0000000000000054 24722475

[B65] Moriarty O. McGuire B. E. Finn D. P. (2011). The effect of pain on cognitive function: a review of clinical and preclinical research. Prog. Neurobiol. 93, 385–404. 10.1016/j.pneurobio.2011.01.002 21216272

[B66] Nilsson L.-G. Nilsson E. (2009). Overweight and cognition. Scand. J. Psychol. 50, 660–667. 10.1111/j.1467-9450.2009.00777.x 19930267

[B67] Okifuji A. Hare B. D. (2011). Do sleep disorders contribute to pain sensitivity? Curr. Rheumatol. Rep. 13, 528–534. 10.1007/s11926-011-0204-8 21805110

[B68] Ong W.-Y. Stohler C. S. Herr D. R. (2019). Role of the prefrontal cortex in pain processing. Mol. Neurobiol. 56, 1137–1166. 10.1007/s12035-018-1130-9 29876878 PMC6400876

[B69] Opdebeeck C. Matthews F. E. Wu Y.-T. Woods R. T. Brayne C. Clare L. (2018). Cognitive reserve as a moderator of the negative association between mood and cognition: evidence from a population-representative cohort. Psychol. Med. 48, 61–71. 10.1017/S003329171700126X 28521844

[B70] Pallarés-Sanmartín A. Gonzalez F. J. C. Fiorentino F. Crespo M. G. (2019). Validation of the sleep Jenkins questionnaire (SJQ) into Spanish: assessment of quality of sleep in patients with asthma. Chest 156:A1715. 10.1016/j.chest.2019.08.1501

[B71] Peña-Casanova J. Casals-Coll M. Quintana M. Sánchez-Benavides G. Rognoni T. Calvo L.. (2012). Spanish normative studies in a young adult population (NEURONORMA young adults project): methods and characteristics of the sample. Neurología (Eng. Ed.) 27, 253–260. 10.1016/j.nrleng.2011.12.008 22397892

[B72] Pertl M. M. Hannigan C. Brennan S. Robertson I. H. Lawlor B. A. (2017). Cognitive reserve and self-efficacy as moderators of the relationship between stress exposure and executive functioning among spousal dementia caregivers. Int. Psychogeriatr. 29, 615–625. 10.1017/S1041610216002337 28067184

[B73] Pessoa L. (2008). On the relationship between emotion and cognition. Nat. Rev. Neurosci. 9, 148–158. 10.1038/nrn2317 18209732

[B74] Pupíková M. Šimko P. Gajdoš M. Rektorová I. (2021). Modulation of working memory and resting-state fMRI by tDCS of the right frontoparietal network. Neural Plast. 2021:5594305. 10.1155/2021/5594305 34349797 PMC8328716

[B75] Rami L. Valls-Pedret C. Bartrés-Faz D. Caprile C. Solé-Padullés C. Castellvi M.. (2011). Cognitive reserve questionnaire. Scores obtained in a healthy elderly population and in one with Alzheimer's disease. Rev. Neurol. 52, 195–201. 10.33588/rn.5204.2010478 21312165

[B76] Reynoso-Alcántara V. Silva-Pereyra J. Fernández-Harmony T. Mondragón-Maya A. (2018). Principales efectos de la reserva cognitiva sobre diversas enfermedades: una revisión sistemática. Psiquiatr. Biol. 25, 53–67. 10.1016/j.psiq.2018.02.005

[B77] Saldanha J. S. Zortea M. Deliberali C. B. Nitsche M. A. Kuo M.-F. Torres I. L.. (2020). Impact of age on tDCS effects on pain threshold and working memory: results of a proof of concept cross-over randomized controlled study. Front. Aging Neurosci. 12:189. 10.3389/fnagi.2020.00189 32714178 PMC7344165

[B78] Sambataro F. Murty V. P. Callicott J. H. Tan H.-Y. Das S. Weinberger D. R.. (2010). Age-related alterations in default mode network: impact on working memory performance. Neurobiol. Aging 31, 839–852. 10.1016/j.neurobiolaging.2008.05.022 18674847 PMC2842461

[B79] Santarnecchi E. Sprugnoli G. Tatti E. Mencarelli L. Neri F. Momi D.. (2018). Brain functional connectivity correlates of coping styles. Cogn. Affect Behav. Neurosci. 18, 495–508. 10.3758/s13415-018-0583-7 29572771

[B80] Scarmeas N. Stern Y. (2003). Cognitive reserve and lifestyle. J. Clin. Exp. Neuropsychol. 25, 625–633. 10.1076/jcen.25.5.625.14576 12815500 PMC3024591

[B81] Seminowicz D. A. Davis K. D. (2006). Cortical responses to pain in healthy individuals depends on pain catastrophizing. Pain 120, 297–306. 10.1016/j.pain.2005.11.008 16427738

[B82] Seminowicz D. A. Moayedi M. (2017). The dorsolateral prefrontal cortex in acute and chronic pain. J. Pain 18, 1027–1035. 10.1016/j.jpain.2017.03.008 28400293 PMC5581265

[B83] Senba E. Kami K. (2017). A new aspect of chronic pain as a lifestyle-related disease. Neurobiol. Pain 1, 6–15. 10.1016/j.ynpai.2017.04.003 31194049 PMC6550110

[B84] Sjøgren P. Christrup L. L. Petersen M. A. Højsted J. (2005). Neuropsychological assessment of chronic non-malignant pain patients treated in a multidisciplinary pain centre. Eur. J. Pain 9, 453–462. 10.1016/j.ejpain.2004.10.005 15979026

[B85] Stern Y. (2002). What is cognitive reserve? Theory and research application of the reserve concept. J. Int. Neuropsychol. Soc. 8, 448–460. 10.1017/S1355617702813248 11939702

[B86] Stern Y. (2009). Cognitive reserve. Neuropsychologia 47, 2015–2028. 10.1016/j.neuropsychologia.2009.03.004 19467352 PMC2739591

[B87] Stern Y. Arenaza-Urquijo E. M. Bartrés-Faz D. Belleville S. Cantilon M. Chetelat G.. (2020). Whitepaper: defining and investigating cognitive reserve, brain reserve, and brain maintenance. Alzheimers Dement 16, 1305–1311. 10.1016/j.jalz.2018.07.219 30222945 PMC6417987

[B88] Suvinen T. I. Kemppainen P. Le Bell Y. Kauko T. Forssell H. (2016). Assessment of pain drawings and self-reported comorbid pains as part of the biopsychosocial profiling of temporomandibular disorder pain patients. J. Oral Facial Pain Head. 30, 287–295. 10.11607/ofph.1589 27792795

[B89] Tamburin S. Maier A. Schiff S. Lauriola M. F. Di Rosa E. Zanette G.. (2014). Cognition and emotional decision-making in chronic low back pain: an ERPs study during Iowa gambling task. Front. Psychol. 5:e01350. 10.3389/fpsyg.2014.01350 25505440 PMC4243494

[B90] Tessitore A. Russo A. Giordano A. Conte F. Corbo D. De Stefano M.. (2013). Disrupted default mode network connectivity in migraine without aura. J. Head. Pain 14:89. 10.1186/1129-2377-14-89 24207164 PMC3832236

[B91] The Whoqol Group (1998). The World Health Organization quality of life assessment (WHOQOL): development and general psychometric properties. Soc. Sci. Med. 46, 1569–1585. 10.1016/S0277-9536(98)00009-4 9672396

[B92] Treede R.-D. Rief W. Barke A. Aziz Q. Bennett M. I. Benoliel R.. (2015). A classification of chronic pain for ICD-11. Pain 156, 1003–1007. 10.1097/j.pain.0000000000000160 25844555 PMC4450869

[B93] Turk D. C. Fillingim R. B. Ohrbach R. Patel K. V. (2016). Assessment of psychosocial and functional impact of chronic pain. J. Pain 17, T21–49. 10.1016/j.jpain.2016.02.006 27586830

[B94] Veldhuijzen D. S. Kenemans J. L. van Wijck A. J. M. Olivier B. Kalkman C. J. Volkerts E. R. (2006). Processing capacity in chronic pain patients: a visual event-related potentials study. Pain 121, 60–68. 10.1016/j.pain.2005.12.004 16480825

[B95] Verbunt J. A. Seelen H. A. Vlaeyen J. W. van de Heijden G. J. Heuts P. H. Pons K.. (2003). Disuse and deconditioning in chronic low back pain: concepts and hypotheses on contributing mechanisms. Eur. J. Pain 7, 9–21. 10.1016/S1090-3801(02)00071-X 12527313

[B96] Villemure C. Schweinhardt P. (2010). Supraspinal pain processing: distinct roles of emotion and attention. Neuroscientist 16, 276–284. 10.1177/1073858409359200 20360603

[B97] Wechsler D. (2012). WAIS-IV. Escala de inteligencia de Wechsler para adultos-IV. Manual de aplicación y corrección. Madrid: NCS Pearson, Inc.

[B98] Wiech K. (2016). Deconstructing the sensation of pain: the influence of cognitive processes on pain perception. Science 354, 584–587. 10.1126/science.aaf8934 27811269

[B99] World Medical Association (2013). World Medical Association declaration of Helsinki: ethical principles for medical research involving human subjects. JAMA 310, 2191–2194. 10.1001/jama.2013.281053 24141714

