# Supplementary material for: Cognitive Reserve as a Protective Factor of Mental Health in Middle-Aged Adults Affected by Chronic Pain
Source: Front Psychol. 2021 Oct 25;12:752623. doi: 10.3389/fpsyg.2021.752623 (PMC8573249; doi:10.3389/fpsyg.2021.752623)
Supplement: Supplementary file 1 [file Table_1.DOCX]

**Supplementary material**

**Cognitive reserve questionnaire**

| **Educational level** | |
| --- | --- |
| None | 0 |
| Read and write autodidacticly | 1 |
| Basic (<6 years) | 2 |
| Elementary School (≥ 6 years) | 3 |
| High school (≥9years) | 4 |
| Diplomacy | 5 |
| **Parent’s educational level** | |
| No | 0 |
| Elementary | 1 |
| Superior | 2 |
| **Training courses** | |
| None | 0 |
| One or two | 1 |
| Between 2 and 5 | 2 |
| More than 5 | 3 |
| **Occupational level** | |
| Not qualified | 0 |
| Unskilled manual labor | 1 |
| Qualified manual labor | 2 |
| Professional (with university studies) | 3 |
| Manager or director (with university studies) | 4 |
| **Musical formation** | |
| Any | 0 |
| Non-regulated training | 1 |
| Regulated musical training | 2 |
| **Languages** | |
| Maternal language | 0 |
| Two languages (including Catalan, Galician, Euskera, Spanish) | 1 |
| Two/three languages (one different to Catalan, Galician, or Euskera) | 2 |
| More than two languages | 3 |
| **Reading activity** | |
| Never | 0 |
| One book a year and/or read the press daily | 1 |
| Between two and five books a year | 2 |
| From 5 to 10 books per year | 3 |
| More than 10 books a year | 4 |
| **Intellectual games** | |
| Never or rarely | 0 |
| Occasionally (1 to 5 /month) | 1 |
| Frequently | 2 |
| **Are you doing any training (courses, music, languages ...)?** | |
| None | 0 |
| One or two | 1 |
| Between two and five | 2 |
| More than five | 3 |
| **How often do you perform cognitively stimulating activities (reading, intellectual games, playing an instrument, painting, writing ...)?** | |
| Never or rarely | 0 |
| Less than once a month | 1 |
| Monthly | 2 |
| Sometimes a month | 3 |
| Weekly | 4 |
| Several times a week | 5 |
| Daily | 6 |
